# *Notes from the Field:* Serum Concentrations of Perfluoroalkyl and Polyfluoroalkyl Substances Among First Responders to the Maui Wildfires — Hawaii, September 2023

**DOI:** 10.15585/mmwr.mm7403a1

**Published:** 2025-02-06

**Authors:** Catherine C. Beaucham, Rachel Zeiler, Kenneth Fent, Sophia K. Chiu, Nicholas Somerville, Alexander Mayer, Jessica L. Rinsky, Cheryl Estill

**Affiliations:** 1 Division of Field Studies and Engineering, National Institute for Occupational Safety and Health, CDC.

**Summary**

**What is already known about this topic?**

Perfluoroalkyl and polyfluoroalkyl substances (PFAS), present in some firefighting foams and routine firefighting activities, can increase the risk for occupational PFAS exposure among firefighters and are associated with cancer and other adverse health outcomes.

**What is added by this report?**

Median summed concentrations of selected PFAS among Maui County, Hawaii employees approximately 1 month after the 2023 Maui wildfires were higher among firefighters than among other responder groups. Sources other than the wildfires could have contributed to the levels.

**What are the implications for public health practice?**

Further research is needed to better understand the occupational risk among groups responding to wildfire disasters. Rostering responders, tracking response activities and personal protective equipment use, and monitoring health could improve the understanding of chemical exposures and guide prevention strategies.

The United States is currently experiencing higher fire activity than in past years with an increase of the acreage burned, and that includes more destructive wildland urban-rural interface fires, potentially exposing first responders and communities to hazardous chemicals in the air and debris (*1*). Exposures to perfluoroalkyl and polyfluoroalkyl substances (PFAS), which are present in fire effluents, PFAS-containing dust, some firefighting foams, protective clothing worn by firefighters, and contaminated gear or equipment, are associated with cancer, cholesterol level changes, and other adverse health outcomes (*2*). Wildfires in Maui, Hawaii in August 2023 destroyed thousands of structures, vehicles, and parcels of land (*3*), and 102 persons lost their lives. Maui County employees (firefighters, police, ocean safety officers, and public works employees) responded and engaged in fire suppression, structure protection, evacuation, water rescue, and urban search and rescue. This report describes first responders’ exposures to PFAS as measures after responding to those fires.

## Investigation and Outcomes

### Request for Assistance

In August 2023, Maui County requested technical assistance from CDC’s National Institute for Occupational Safety and Health (NIOSH) to evaluate first responders’ exposures to selected chemicals during the 2023 Maui wildfires, through a mission assignment from the Federal Emergency Management Agency. In September 2023 (approximately 1 month after the initial wildfire response), NIOSH medical and exposure assessment personnel and a CDC logistician traveled to Maui County to evaluate potential exposures in firefighters and other responders.

### Data Collection

All Maui County employees who were involved in the first 5 days (August 8–12, 2023) of the wildfire response were invited to participate in the evaluation. NIOSH collected spot urine and blood specimens and demographic and workplace information via questionnaires. Biologic specimens were analyzed at CDC’s National Center for Environmental Health Division of Laboratory Sciences. Among other chemicals, PFAS were selected as analytes, because they can be expected in the wildland urban-rural interface environment and have relatively long elimination half-lives, which can facilitate detection in serum weeks after an event. This activity was reviewed by CDC, deemed not research, and was conducted consistent with applicable federal law and CDC policy.*

### Data Analysis

Individual serum PFAS concentrations were compared with the National Health and Nutrition Examination Survey (NHANES) 95th percentile concentrations for persons aged ≥20 years during survey years 2017–2018.^†^ The sum of seven PFAS was compared with the National Academies of Sciences, Engineering, and Medicine (NASEM) clinical threshold, above which NASEM recommends that clinicians prioritize screening for cancer and other adverse health outcomes.^§^ ANOVA of log-transformed continuous values was used to compare levels between occupational subgroups. R (version 4.3.3; R Foundation) statistical software was used to conduct analyses.

In total, 258 Maui County employees, including 178 (69%) firefighters, submitted blood samples. Significant differences in concentrations of perfluorohexane sulfonic acid (PFHxS), one of the most biologically persistent PFAS examined, were identified by occupational subgroup (p<0.01), with the highest concentrations detected among firefighters (median = 1.2 *μ*g/L; [IQR = 0.8–1.7 *μ*g/L]). The highest detected PFHxS concentration (9.3 *μ*g/L) was in a firefighter; this level was approximately 2.5 times the NHANES 95th percentile (3.8 *μ*g/L) while levels of PFHxS for the other participants range from not detectable to 3.8 *μ*g/L. For the other PFAS chemicals, 2–40% of all Maui County employees, including firefighters, were over the NHANES 95th percentile.

Among firefighters, the median sum serum concentrations of seven PFAS was 7.0 *μ*g/L (Figure). The firefighter with the highest serum PFHxS concentration also had a summed serum PFAS concentration above the NASEM clinical threshold of 20 *μ*g/L. This firefighter was advised to consult a health care provider for additional medical screening and follow-up. All other Maui County participants were below the clinical threshold. The median sum serum concentrations of seven PFAS for other occupational subgroups ranged from 5.7 *μ*g/L to 6.9 *μ*g/L. In general, higher median summed PFAS concentrations were detected among firefighters with longer job tenure (≥30 years) than in those with shorter tenures, although multiple outliers were present in the <5 years of work category (Figure). For longer-term surveillance, firefighters were offered the opportunity to enroll in the National Firefighter Registry for Cancer, which provides long-term tracking of cancer outcomes.^¶^

**FIGURE:**
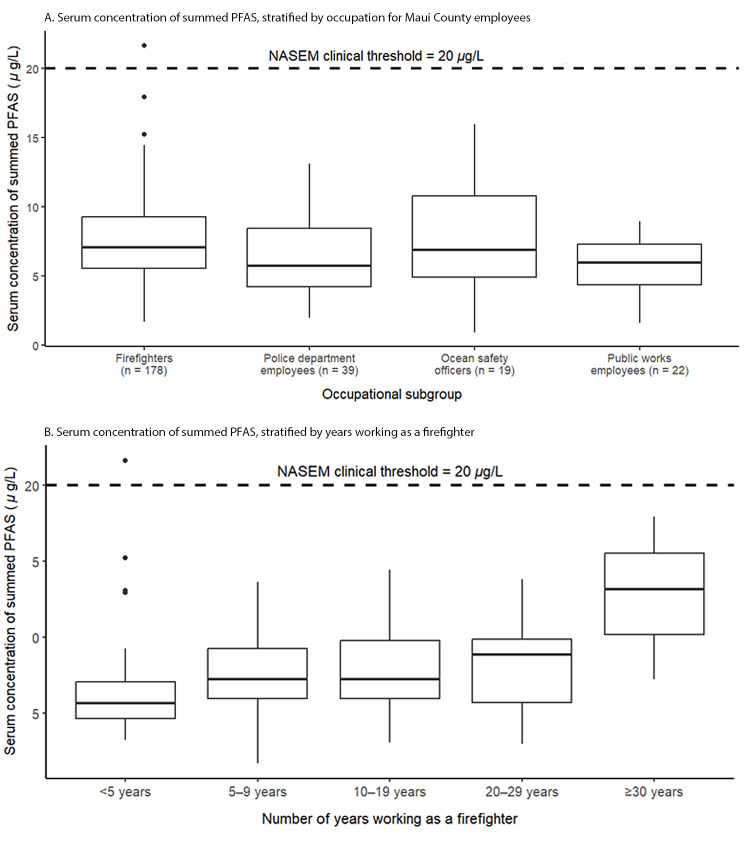
Serum concentrations* of the sum of seven perfluoroalkyl and polyfluoroalkyl substances in (A) all county employees (N = 258), by occupation, and (B) firefighters (N = 178), by number of years working in the profession^†^ — Maui County, Hawaii, September 2023 **Abbreviations:** NASEM = National Academies of Sciences, Engineering, and Medicine; PFAS = perfluoroalkyl and polyfluoroalkyl substances. * Median values represented by the center horizonal line of each box; top and bottom lines represent the IQR, the vertical lines represent the minimum and maximum values that fall into 1.5 times the IQR, and the dots represent outliers that are more or less than 1.5 times the IQR. **^†^** Firefighters only (N = 178).

The median age of the 259 Maui County employees who responded to the questionnaire was 40.0 years (range = 20.6–68.7 years), and 94% were male. Respondents, who could select multiple racial groups, most commonly identified as non-Hispanic White (54%), non-Hispanic Asian (48%), or non-Hispanic Native Hawaiian or other Pacific Islander (46%); 7% identified as non-Hispanic American Indian or Alaska Native, and 1% as non-Hispanic Black or African American. Reported use of respiratory protection varied widely during the response, according to activities performed and among occupational subgroups, and was more commonly used by those who responded to active fires (approximately 40% of firefighters) than those who did not (4% to 33% of other responders). 

## Preliminary Conclusions and Actions

Sources of PFAS exposure for firefighters include fire effluents and dust containing PFAS, PFAS contamination on gear or equipment, PFAS in textiles that constitute the protective clothing worn by firefighters, or PFAS-containing firefighting foams (*2*). Previous studies have shown that firefighters have elevated serum concentrations of some PFAS, including PFHxS, compared with the general population and other working populations (*4*). Large wildfire disasters that encroach upon urban areas present a challenging environment for first responders.

Firefighters in this assessment had higher serum concentrations of some PFAS than employees from other occupations, but sum serum concentrations were mostly below a recommended threshold indicating the need to perform additional medical screenings. Because specimen collection occurred approximately 1 month after the initial response without any baseline measurements, the relative contribution of PFAS from the 2023 Maui wildfires compared with other sources is unclear. Inconsistent use of respirators and other personal protective equipment could increase the risk for exposure to PFAS and other chemicals. In July 2024, NIOSH published a Health Hazard Evaluation Report summarizing the comprehensive biologic monitoring results and recommendations for limiting exposures (*5*). These findings are useful to advancing understanding of health implications and guiding public health decision-making for ongoing and future fires. To protect first responders during disasters, it is essential to continue providing strategies for characterizing and reducing exposures, including through the rostering of responders, tracking response activities and use of personal protective equipment, and health monitoring, such as implementing the Emergency Responder Health Monitoring and Surveillance framework.**

## Footnotes

* 45 C.F.R. part 46.102(l)(2), 21 C.F.R. part 56; 42 U.S.C. Sect. 241(d); 5 U.S.C. Sect. 552a; 44 U.S.C. Sect. 3501 et seq.

†
https://www.cdc.gov/environmental-exposure-report/data-tables/index.html

§ The NASEM clinical threshold is the sum of seven PFAS ≥20 *μ*g/L, above which NASEM recommends that clinicians prioritize additional screenings (i.e., for cancer, cholesterol level changes, and other adverse health outcomes) in addition to the usual standard of care. NASEM states that concentrations between 2–20 *μ*g/L are associated with possible adverse effects, especially in sensitive populations. The seven PFAS were PFHxS, perfluorooctane sulfonic acid (PFOS), perfluorooctanoic acid (PFOA), perfluorononanoic acid (PFNA), perfluorodecanoic acid (PFDA), perfluoroundecanoic acid (PFUnDA), and 2-(n-methyl-perfluorooctane sulfonamido) acetic acid (MeFOSAA). https://nap.nationalacademies.org/resource/26156/PFAS%20Guidance%20Highlights.pdf

¶ The National Firefighter Registry for Cancer is the largest effort undertaken by the nation to support and advance understanding of cancer in the fire service. While participation is voluntary, all U.S. firefighters, with or without cancer, are encouraged to join the National Firefighter Registry for Cancer. https://www.cdc.gov/niosh/firefighters/registry/index.html

## References

[1] Environmental Protection Agency. Climate change indicators: wildfires. Washington, DC: Environmental Protection Agency; 2024. https://www.epa.gov/climate-indicators/climate-change-indicators-wildfires

[2] National Institute for Occupational Safety and Health. PFAS and worker health. Cincinnati, OH: US Department of Health and Human Services, CDC, National Institute for Occupational Safety and Health; 2024. https://www.cdc.gov/niosh/pfas/about/index.html

[3] Environmental Protection Agency. Maui wildfires. Washington, DC: Environmental Protection Agency; 2024. https://www.epa.gov/maui-wildfires

[4] Christensen BT, Calkins MM. Occupational exposure to per- and polyfluoroalkyl substances: a scope review of the literature from 1980–2021. J Expo Sci Environ Epidemiol 2023;33:673–86. 10.1038/s41370-023-00536-y 36977833 PMC10533727

[5] Somerville N, Beaucham CC, Mayer AC, Zeiler RJ, Estill CF, Fent K. Evaluation of first responders’ biological monitoring results after Maui County Hawaii wildfires: HHE Report Nos. 2023-0136 and 2023-0142-3400. Cincinnati, OH: US Department of Health and Human Services, CDC, National Institute for Occupational Safety and Health; 2024. https://www.cdc.gov/niosh/hhe/reports/pdfs/2023-0136-0142-3400.pdf

